# Onchocerciasis drug development: from preclinical models to humans

**DOI:** 10.1007/s00436-021-07307-4

**Published:** 2021-10-13

**Authors:** Adela Ngwewondo, Ivan Scandale, Sabine Specht

**Affiliations:** 1 Centre of Medical Research, Institute of Medical Research and Medicinal Plants Studies (IMPM), P.O. Box13033, Yaoundé, Cameroon; 2 Drugs for Neglected Diseases Initiative, Chemin Camille-Vidart 15, 1202 Geneva, Switzerland

**Keywords:** Onchocerciasis, Macrofilaricide, Microfilaricide, Preclinical models, Clinical trials, Mode of action, Drug development

## Abstract

Twenty diseases are recognized as neglected tropical diseases (NTDs) by World Health Assembly resolutions, including human filarial diseases. The end of NTDs is embedded within the Sustainable Development Goals for 2030, under target 3.3. Onchocerciasis afflicts approximately 20.9 million people worldwide with > 90% of those infected residing in Africa. Control programs have made tremendous efforts in the management of onchocerciasis by mass drug administration and aerial larviciding; however, disease elimination is not yet achieved. In the new WHO roadmap, it is recognized that new drugs or drug regimens that kill or permanently sterilize adult filarial worms would significantly improve elimination timelines and accelerate the achievement of the program goal of disease elimination. Drug development is, however, handicapped by high attrition rates, and many promising molecules fail in preclinical development or in subsequent toxicological, safety and efficacy testing; thus, research and development (R&D) costs are, in aggregate, very high. Drug discovery and development for NTDs is largely driven by unmet medical needs put forward by the global health community; the area is underfunded and since no high return on investment is possible, there is no dedicated drug development pipeline for human filariasis. Repurposing existing drugs is one approach to filling the drug development pipeline for human filariasis. The high cost and slow pace of discovery and development of new drugs has led to the repurposing of “old” drugs, as this is more cost-effective and allows development timelines to be shortened. However, even if a drug is marketed for a human or veterinary indication, the safety margin and dosing regimen will need to be re-evaluated to determine the risk in humans. Drug repurposing is a promising approach to enlarging the pool of active molecules in the drug development pipeline. Another consideration when providing new treatment options is the use of combinations, which is not addressed in this review. We here summarize recent advances in the late preclinical or early clinical stage in the search for a potent macrofilaricide, including drugs against the nematode and against its endosymbiont, *Wolbachia pipientis.*

## Onchocerciasis

Onchocerciasis remains a leading cause of debilitating skin and ocular disease in endemic regions in Africa and the Arabian Peninsula. It is a vector-borne parasitic disease caused by the filarial nematode *Onchocerca volvulus* (Burnham 1998). Arthropod vectors (*Simulium spp.*) acquire L1 larval stages (microfilariae, mf) during a blood meal from the human host; L1 larvae subsequently moult twice to the L3 (infective) stage in the vector and are then introduced into a new host during a blood meal where they subsequently develop to adult stages. L3 larvae moult inside the human host to L4 and then L5 stages before maturing to adults over a period of months. In the human body, adult worms produce larvae (microfilariae) that migrate to the skin, eyes and other organs. When a female blackfly bites an infected person during a blood meal, it ingests the skin-dwelling microfilariae which develop and are subsequently transmitted to the next human host.

A subcutaneous nodule is formed around the female worms, the core of which is a dense infiltrate of inflammatory cells. Male adults migrate between nodules, where they fertilize sedentary females. After mating, females release thousands of microfilariae into the surrounding tissues, where they can be ingested by feeding black flies to complete the life cycle.

Most clinical manifestations associated with *O. volvulus* are related to the chronic effects of repeated episodes of inflammation induced by the death of microfilariae and most likely also their *Wolbachia* endosymbionts. Skin disease is caused by inflammation around skin-dwelling microfilariae; it presents primarily as a generalized papular dermatitis and rarely as hyperreactive localized onchodermatitis (“sowda”). Infection with *O. volvulus* can cause visual impairment and blindness, including anterior segment disease with sclerosing keratitis and iridocyclitis, and posterior segment disease with optic atrophy and chorioretinopathy (Nutman 2020).

Microfilariae have been targeted by chemotherapeutic mass treatment with ivermectin (IVM), which is currently the most important drug for the treatment of onchocerciasis. It is well tolerated, highly efficacious and rapidly reduces microfilarial numbers in the skin (Aziz et al. 1982). Ivermectin significantly reduces the itching of reactive onchocercal skin disease (Brieger et al. 1998); however, because microfilariae can re-invade the skin within 6 months of treatment, annual treatments may not be sufficient to control pruritus, and treatment every 3 months may be necessary for the first 1 to 2 years. Sub-optimal efficacy has been observed in patients endemic in areas repeatedly treated with ivermectin (Osei-Atweneboana et al. 2007).

The Global Burden of Disease Study in 2017 estimated that 20.9 million people are infected with *O. volvulus* worldwide; 14.6 million of those infected suffer from skin disease and 1.15 million have experienced vision loss (Global Burden of Disease 2018).

## Current treatment strategy

The initial effort of the West African Onchocerciasis Control Program (OCP) to reduce disease, which focused on eliminating the vector using aerial larvaciding, was greatly enhanced in 1987 by preventive chemotherapy (PC) with ivermectin donated by Merck & Co. In 1995, the use of ivermectin for onchocerciasis was extended to cover all endemic countries in Africa with the establishment of the African Programme for Onchocerciasis Control (APOC). APOC was formed with the extended mandate of reducing the prevalence of both blinding and dermatological disease by distributing ivermectin on an annual basis to all (above 5 years of age, not pregnant) living in meso-and hyper-endemic communities. With the closure of APOC in 2015, onchocerciasis elimination efforts across Africa are now under the auspices of the Expanded Special Project for Elimination of Neglected Tropical Diseases (ESPEN) with the aim for disease control as a “public health problem” shifting now to “elimination of transmission”, as formulated in the new World Health Organisation (WHO) NTD Roadmap (WHO 2020).

Four out of six countries in the Americas have been verified by WHO as free of onchocerciasis after successful implementation of elimination activities: Colombia, Ecuador, Mexico and Guatemala (Sauerbrey et al. 2018). The regional focus is now on the remaining active transmission zone, called the Yanomami Area, on the border between Venezuela and Brazil. Both countries have difficult political climates that hinder the elimination task in this remote and neglected region.

Elimination of transmission has been also achieved in focal areas of Africa (e.g. Sudan, Uganda and Ethiopia (Global Burden of Disease 2018). The global effort against onchocerciasis has been one of the most successful public health initiatives in tropical medicine of the past century. To ensure continuous success and hinder recrudescence in any of these sites, surveillance studies are indispensable.

## Limitations of current MDA strategies

The success and effectiveness of Mass Drug Administration (MDA) are highly dependent on treatment coverage and pre-control endemicity (Turner et al. 2014; Coffeng et al. 2014; Stolk et al. 2015). An effective, affordable and feasible way forward is under continual discussion, and several limitations of the MDA programme have been identified.

Onchocerciasis foci have been classified epidemiologically, according to infection prevalence, into hyper-endemic (> 60%), mesoendemic (30–60%) and hypo-endemic (< 30%). Previously, hyper- and mesoendemic areas were targeted, but not hypo-endemic areas. With the major shift taking place in the last few years to move from the control of the disease as a “public health problem” to “elimination of transmission”, hypo-endemic areas are now also included, as they remain a source of ongoing infection. One of the greatest challenges facing this new goal is the need to remap endemic areas of onchocerciasis to allow optimal IVM distribution.

Another consideration is that coinfection with *Loa loa* hinders elimination efforts in countries such as Nigeria, Cameroon, Ethiopia, Uganda and Congo (WHO 2014) because treatment with IVM of persons with high-level *Loa loa* microfilaremia can trigger serious complications, including meningoencephalitis (Boussinesq et al. 2003). These areas were previously excluded from MDA, making the coverage of current MDA programs incomplete. A new strategy termed “Test and Not Treat” (TaNT), based on the use of the LoaScope to estimate *L. loa* microfilaraemia before treatment to exclude heavily infected patients, could allow safe implementation of MDA in loiasis endemic areas (Lenk et al. 2020; Boussinesq et al. 2018). However, this has not yet been implemented on a large scale.

Non-compliance has been another major issue during MDA campaigns. Although not analysed systematically, post-treatment adverse events have been a major reason for refusing IVM intake. This was systematically addressed in a recent study in a *Loa loa*-coendemic area (Forrer et al. 2021). Furthermore, the reasons for non-compliance were analysed in two health districts in the western region of Cameroon. Disease prevalence remained high in these areas, despite the annual distribution of IVM since 1996. Nearly 30% of the population did not take IVM during the most recent round of MDA and there was a significant proportion of the population that had reportedly never taken the drug (Katabarwa et al. 2013). The key factors associated with drug adherence were related to either programmatic and delivery issues, primarily absenteeism at the time of the campaign, or individual determinants, such as side effects associated with the drug, ethnicity, age and years lived in the village. Efforts should also include social scientists in control programs to better understand and reduce the systematic non-compliance in certain groups are likely to be important in ensuring the interruption of transmission in the study area (Senyonjo et al. 2016).

After years of implementation, MDA programme fatigue should also be considered, as individuals in an endemic area may find repeated MDA inconvenient or may lose confidence in the MDA campaign. The benefits of effective MDA for the entire population clearly exceed the risk, but when prevalence is reduced, the risk/benefit ratio may narrow considerably. Unlike a vaccine, MDA for onchocerciasis does not provide an immediate benefit for an uninfected person, potentially leading to an additional lack of treatment adherence.

Other equally important factors include areas being difficult to treat due to ongoing conflict, lack of financial resources and inadequate political engagement, for example in the Democratic Republic of Congo (Makenga et al. 2015).

Finally, obtaining up-to-date data on remaining transmission zones and monitoring elimination efforts are critical to the success of the global programme for onchocerciasis control, as the accurate mapping will allow targeted interventions and provide the information needed to guide decisions on when to stop MDA. To do this, improved diagnostic tests are urgently needed to fine-tune elimination efforts.

In April 2020, the WHO published a new roadmap for NTDs for the period 2021–2030, which aims to guide countries towards achieving Sustainable Development Goal 3.3, to ensure healthy lives and promote well-being for all at all ages and to end endemic NTDs by 2030. It proposes important shifts for NTD programs, which will be fundamental to sustaining the progress in control of NTDs, including filarial diseases. The need for strong country ownership, including domestic funding, and a more holistic approach to address cross-cutting development issues has been recognized, and programs and activities to fight NTDs should be further combined across diseases to achieve a maximum return on investment. It has also been recognized that additional tools, including the development of a macrofilaricide as an alternative strategy (WHO 2020; Walker et al. 2017a), are needed to significantly reduce the timelines for elimination as part of the strategy (NTD Modelling Consortium Onchocerciasis Group. Gates Open Res. 2019).

## Drug discovery in onchocerciasis

Before any new drug is tested in clinical trials, its safety and efficacy must be evaluated through streamlined processes and well-established tests. Assessment of the preclinical safety profile follows clear harmonized guidelines, while a demonstration of efficacy is far more complicated. In vitro and in vivo models serve to demonstrate efficacy against the whole parasite and/or specific molecular targets (protein, RNA molecule, etc.) Drug discovery in veterinary parasitology, therefore, consists of two complementary lead identification strategies and includes phenotypic screening (whole organism) and target-based screening (enzymes, receptors). Whereas target-based screens benefit from technological advances in structural biology, computational chemistry, structure-based drug design, genomics and proteomics, coupled with enhanced automation in high-throughput screening platforms and combinatorial chemistry strategies, most antiparasitic products were discovered by the phenotypic screening of synthetic and natural compounds against intact/whole parasites, either in culture or in animal models (Selzer and Epe 2020). Because of limited resources available for human NTDs, drug discovery relies predominantly on phenotypic screening and only a few biological targets have been described and validated.

In veterinary drug discovery, molecules can be evaluated in infection models matching the pathologies of the animals which are the final beneficiaries. As human pathogens are often not viable in animal hosts, drug discovery for humans relies on surrogate parasites for in vitro and in vivo assays. Therefore, the trickiest question in drug development for filarial parasites has always been: which is the most predictive model for selecting a drug candidate and predicting efficacy in humans? The simple answer is that there isn’t one. Every host-parasite combination has its own strengths and limitations. Foremost, the larval biology of filarial worms presents common, but also clearly distinct, features, such as the location of the parasite, the parasite-host interaction and clinical presentation within the host. To select novel and effective anti-filarial drugs, all available information (activity in vitro against different worm stages, activity in different in vivo models, pharmacokinetic parameters in the final hosts, etc.) should be carefully reviewed, as none of the existing models fully reflects the situation in human filarial infections. These aspects must be considered when interpreting an animal model for testing or predicting drug efficacy.

The standards of care for several filarial infections, IVM and diethylcarbamazine (DEC) for lymphatic filariasis, are potent inhibitors of microfilaremia which have been remarkably successful in reducing transmission and clinical symptoms in humans. It is, however, important to note that although these molecules are ineffective in vitro at relevant clinical concentrations, they are efficacious in animal models of filariasis. Such a disconnect with reference drugs hinders the setup of a straightforward “one-fits-all” screening cascade. Many publications report the parallel testing of a drug in different rodent models to check for consistency of activity across models. However, in the absence of a clinically validated macrofilaricidal treatment, the translation of all these models remains unknown. Therefore, a sensible approach to deciding whether to move a compound forward should be based on the identification of the pharmacokinetic/pharmacodynamic relationship and the mechanism of action. This information is indeed crucial to guiding clinical development and to predicting regimens with reasonable efficacy in humans.

*Onchocerca volvulus* has recently been successfully established for in vitro testing (Voronin et al. 2019; Gandjui et al. 2021) but maintaining it under experimental conditions requires a great effort, and no permissive animal model exists. The infection of fully immunocompetent BALB/c mice with infective third-stage *L. sigmodontis* larvae (L3) results in patent infections with circulating microfilariae. Alternative surrogate models are *Brugia pahangi* (host: rodent, human), *Acanthochieloma viteae* (host: jirds), *Onchocerca gutturosa* (host: cattle), *Onchocerca linealis* (host: cattle) and *O. Ochengi* (host: cattle) (Morris et al. 2013). Recent attempts tried to overcome this problem by using transgenic rodent hosts, modified to tolerate human filarial species (Patton et al. 2018; Pionnier et al. 2020).

Intracellular bacteria in filarial nematodes were discovered in the 1970s with the advent of electron microscopy. It was speculated that these bacteria were related to insect *Wolbachia* symbionts and suggested that these bacteria might contribute to the pathogenesis of the filarial disease, making them a novel target for anti-filarial chemotherapy (reviewed in Taylor and Hoerauf 1999). In the beginning of the 2000s, *Wolbachia* resurfaced as a promising target for the treatment of human filariasis (reviewed in Taylor et al. 2005). Depletion of *Wolbachia* results in the inhibition of embryogenesis and thus a slow decline of circulating microfilariae, followed by adult worm death (Hoerauf et al. 2008); this has been shown using tetracyclines in humans (Table 1) as well as in animal models (Bosshardt et al. 1993; Volkmann et al. 2003; Walker et al. 2015; Aljayyoussi et al. 2017) (Table 2). In contrast to the difficulties described for direct-acting drugs, the identification of this indirect mode of action by *Wolbachia* depletion has enabled the setting up of an efficient screening cascade to allow for high-throughput screening of anti-bacterial drugs (Johnston et al. 2014).

**Table 1:** Major clinical trials in onchocerciasis drug development

	Drug name	Study title	Clinical trial identifier	Reference
**Direct-acting drugs**				
1	Ivermectin	Efficacy and tolerance of ivermectin in human onchocerciasis	None	Aziz et al. (1982)
		Management in Senegal of the 1st efficacy and tolerability studies of ivermectin (MK 933) in human onchocerciasis		Diallo et al. (1984)
		The chemotherapy of onchocerciasis X. An assessment of four single dose treatment regimes of MK-933 (ivermectin) in human onchocerciasis	None	Awadzi et al. (1985)
		Comparison of ivermectin and diethylcarbamazine in the treatment of onchocerciasis	None	Greene et al. (1985)*
		Double-blind study of ivermectin and diethylcarbamazine in African onchocerciasis patients with ocular involvement	None	Lariviere et al. (1985)*
		A double-blind comparison of the efficacy and safety of ivermectin and diethylcarbamazine in a placebo-controlled study of Senegalese patients with onchocerciasis	None	Diallo et al. (1986)*
		The chemotherapy of onchocerciasis. XI. A double-blind comparative study of ivermectin, diethylcarbamazine and placebo in human onchocerciasis in northern Ghana	None	Awadzi et al. (1986)*
		Treatment of onchocerciasis. The ocular effects of ivermectin and diethylcarbamazine	None	Taylor et al. (1986)
		Controlled trial and dose-finding study of ivermectin for treatment of onchocerciasis	None	White et al. (1987)*
		Ocular findings in a double-blind study of ivermectin versus diethylcarbamazine versus placebo in the treatment of onchocerciasis	None	Dadzie et al. (1987)
		Ivermectin effect on microfilariae of *Onchocerca volvulus* after a single oral dose in humans	None	Soboslay et al. (1987)
		Ivermectin in the treatment and prevention of human onchocerciasis	None	Lariviere et al. (1987)
		Effect of single-dose ivermectin therapy on human *Onchocerca volvulus* infection with onchocercal ocular involvement	None	Newland et al. (1988)
		Ivermectin and human onchocerciasis. A study of 234 onchocerciasis patients in the Republic of Mali	None	Vingtain et al. (1988)
		Emergence of *Onchocerca volvulus* microfilariae from skin snips before and after treatment of patients with ivermectin	None	Mössinger et al. (1988)
		A multi-centre study of the effect of Mectizan treatment on onchocercal skin disease: clinical findings	None	Ogbuagu and Eneanya (1988)
		Studies with ivermectin in onchocerciasis patients in northern Ghana, a region with long lasting vector control	None	Awadzi et al. (1989)
		Ivermectin treatment of patients with severe ocular onchocerciasis	None	Taylor et al. (1989)
		Ophthalmological results from a placebo controlled comparative 3-dose ivermectin study in the treatment of onchocerciasis	None	Dadzie et al. (1989)
		A study in the Ivory Coast (1985–1987) of the efficacy and tolerance of ivermectin (Mectizan) in human onchocerciasis. I. A comparative double-blind study of 220 patients with onchocerciasis treated with a single oral dose of 100, 150 or 200 mcg/kg	None	Lariviere et al. (1989)
		Effects of diethylcarbamazine and ivermectin on the mobilization of microfilariae of *Onchocerca volvulus*	None	Basset et al. (1989)
		Lack of adverse reactions in ivermectin treatment of onchocerciasis	None	De Sole et al. (1990)
		Effects of multiple monthly doses of ivermectin on adult *Onchocerca volvulus*	None	Duke et al. (1990)
		Viability of adult *Onchocerca volvulus* after six 2-weekly doses of ivermectin	None	Duke et al. (1991a)
		Comparison of the effects of a single dose and of four six-monthly doses of ivermectin on adult Onchocerca volvulus	None	Duke et al. (1991b)
		A community trial of ivermectin for onchocerciasis in Sierra Leone: adverse reactions after the first five treatment rounds	None	Whitworth et al. (1991a)
		Effects of repeated doses of ivermectin on ocular onchocerciasis: community-based trial in Sierra Leone	None	Whitworth et al. (1991b)
		A comparison of 6-, 12-, and 24-monthly dosing with ivermectin for treatment of onchocerciasis	None	Greene et al. (1991)
		Effects of three-month doses of ivermectin on adult *Onchocerca volvulus*	None	Duke et al. (1992)
		A community trial of ivermectin for onchocerciasis in Sierra Leone: clinical and parasitological responses to four doses given at six-monthly intervals	None	Whitworth et al. (1992a)
		Ivermectin does not reduce the burden of itching in an onchocerciasis endemic community	None	Whitworth et al. (1992b)
		Tolerance of ivermectin treatment of rural communities infected by savannah onchocerciasis in Mali	None	Soula et al. (1992)
		The effect of repeated doses of ivermectin on adult female *Onchocerca volvulus* in Sierra Leone	None	Chavasse et al. (1992)
		Reduction in incidence of optic nerve disease with annual ivermectin to control onchocerciasis	None	Abiose et al. (1993)
		Adverse reactions to ivermectin treatment for onchocerciasis. Results of a placebo-controlled, double-blind trial in Malawi	None	Burnham (1993)
		A community-based trial of ivermectin for onchocerciasis control in the forest of southwestern Cameroon: clinical and parasitologic findings after three treatments	None	Somo et al. (1993)
		Community treatment with ivermectin for onchocerciasis in the east Usambara mountains	None	Mwetta and Hills (1994)
		A trial of a three-dose regimen of ivermectin for the treatment of patients with onchocerciasis in the UK	None	Churchill et al. (1994)
		Transient changes in cytokine profiles following ivermectin treatment of onchocerciasis	None	Steel et al. (1994)
		Ivermectin and onchocercal optic neuritis: short-term effects	None	Murdoch et al. (1994)
		The chemotherapy of onchocerciasis. XIX: The clinical and laboratory tolerance of high dose ivermectin	None	Awadzi et al. (1995)
		Ivermectin treatment of onchocercal skin lesions: observations from a placebo-controlled, double-blind trial in Malawi	None	Burnham (1995)
		Effets secondaires du traitement de la loase hypermicrofilarémique par l’ivermectine [Secondary effects of the treatment of hypermicrofilaremic loiasis using ivermectin]	None	Ducorps et al. (1995)
		Irreversible effects of ivermectin on adult parasites in onchocerciasis patients in the Onchocerciasis Control Programme in West Africa	None	Plaisier et al. (1995)
		The effects of multiple doses of ivermectin on ocular onchocerciasis. A six-year follow-up	None	Mabey et al. (1996)
		A community trial of ivermectin for onchocerciasis in Sierra Leone: compliance and parasitological profiles after three and a half years of intervention	None	Whitworth et al. (1996a)
		Clinical and parasitological responses after up to 6.5 years of ivermectin treatment for onchocerciasis	None	Whitworth et al. (1996b)
		An improved dosing schedule for ivermectin as a microfilaricidal agent against onchocerciasis	None	Shu et al. (1997)
		Impact of annual dosing with ivermectin on progression of onchocercal visual field loss	None	Cousens et al. (1997)
		The effects of ivermectin on onchocercal skin disease and severe itching: results of a multicentre trial	None	Brieger et al. (1998)
		The effects of high-dose ivermectin regimens on *Onchocerca volvulus* in onchocerciasis patients	None	Awadzi et al. (1999)
		Effect of repeated ivermectin treatments on ocular onchocerciasis: evaluation after six to eight doses	None	Chippaux et al. (1999)
		A controlled prospective trial of the prophylactic effect of a single dose of ivermectin against *Onchocerca volvulus*	None	Boussinesq and Chippaux (2001)
		Immunocompetence may be important in the effectiveness of Mectizan (ivermectin) in the treatment of human onchocerciasis	None	Ali et al. (2002)
		Effects of standard and high doses of ivermectin on adult worms of *Onchocerca volvulus:* a randomised controlled trial	None	Gardon et al. (2002)
		Thirty-month follow up of sub-optimal responders to multiple treatments with ivermectin, in two onchocerciasis endemic foci in Ghana	None	Awadzi et al. (2004)
		Adverse systemic reactions to treatment of onchocerciasis with ivermectin at normal and high doses given annually or three-monthly	None	Kamgno et al. (2004)
		A randomized, double-blind, controlled trial of the effects of ivermectin at normal and high doses, given annually or three-monthly, against *O. volvulus*: ophthalmological results	NCT02511353	Fobi et al. (2005)
		Chemokines in onchocerciasis patients after a single dose of ivermectin	None	Fendt et al. (2005)
		Evidence for macrofilaricidal activity of ivermectin against female *Onchocerca volvulus*: further analysis of a clinical trial in the Republic of Cameroon indicating two distinct killing mechanisms	None	Duke (2005)
		Non-adherence to community directed treatment with ivermectin for onchocerciasis control in Rungwe district, southwest Tanzania	None	Lakwo and Gasarasi. (2006)
		Ivermectin dose assessment without weighing scales	None	Alexander et al. (1993)
		Individual host factors associated with *Onchocerca volvulus* microfilarial densities 15, 80 and 180 days after a first dose of ivermectin	None	Pion et al. (2011)
		Sustainable control of onchocerciasis: ocular pathology in onchocerciasis patients treated annually with ivermectin for 23 years: a cohort study	PACTR201303000464219	Banla et al. (2014)
		Macrofilaricidal efficacy of repeated doses of ivermectin for the treatment of river blindness	None	Walker et al. (2017b)
		Safety and pharmacokinetic profile of fixed dose ivermectin with an innovative 18-mg tablet in healthy adult volunteers	NCT03173742	Muñoz et al. (2018)
		Single versus multiple dose ivermectin regimen in onchocerciasis-infected persons with epilepsy treated with Phenobarbital: A randomized clinical trial in the Democratic Republic of Congo	NCT03052998	Mandro et al. (2020)
		Ivermectin as an adjuvant to anti-epileptic treatment in persons with onchocerciasis associated epilepsy: a randomized proof-of-concept clinical trial	NCT03052998	Mandro et al. (2020)
		Comparison of repeated doses of ivermectin versus ivermectin plus albendazole for the treatment of onchocerciasis: a randomized, open-label, clinical trial	ISRCTN50035143	Debrah et al. (2020)
		Safety and efficacy of IDA for onchocerciasis (Dolf IDA/Oncho)	NCT04188301	Study ongoing
		Efficacy of ivermectin and albendazole against onchocerciasis in the Volta Region, Ghana	NCT02078024	Study ongoing
		An open study of ivermectin at 150 mcg/kg single dose, capsule formulation) for the treatment of onchocerciasis in children 6–13 years old. 6 months follow up	None	Not published
2	Moxidectin	The antiparasitic moxidectin: safety, tolerability, and pharmacokinetics in humans	None	Cotreau et al. (2003)
		Excretion of moxidectin into breast milk and pharmacokinetics in healthy lactating women	None	Korth-Bradley et al. (2011)
		Relative bioavailability of liquid and tablet formulations of the antiparasitic moxidectin	None	Korth-Bradley et al. (2012)
		A randomized, single-ascending-dose, ivermectin-controlled, double-blind study of moxidectin in *Onchocerca volvulus* infection	NCT00300768	Awadzi et al. (2014)
		Single dose moxidectin versus ivermectin for *Onchocerca volvulus* infection in Ghana, Liberia and the Democratic Republic of the Congo; a randomized, controlled, double-blind phase 3 trial	NCT00790998	Opoku et al. (2018)
		Safety of a single dose of moxidectin compared with ivermectin in individuals living in onchocerciasis endemic areas	NCT04311671	Recruiting
		Safety and efficacy of annual or biannual doses of moxidectin or ivermectin for onchocerciasis	NCT03876262	Recruiting
		A pharmacokinetic and safety study of moxidectin to identify an optimal dose for treatment of children 4 to 11 years	NCT03962062	Recruiting
3	Emodepside	First in man clinical trial of emodepside (BAY 44–4400)	NCT02661178	Gillon et al. (2021)
		Safety, tolerability and pharmacokinetics of emodepside, a potential novel treatment for onchocerciasis (river blindness), in healthy male subjects	NCT03383614	Gillon et al. (2021)
		Relative bioavailability study of emodepside IR-tablets and solution	NCT03383523	Not yet published
		Emodepside phase II for the treatment of onchocerciasis	PACTR202010898529928	Not yet started
4	Oxfendazole	Evaluating the safety and pharmacokinetics of oxfendazole	NCT02234570	An et al. (2019)
		Pharmacokinetics, safety, and tolerability of oxfendazole in healthy adults in an open-label phase 1 multiple ascending dose and food effect study	NCT03035760	Bach et al. (2020)
5	Auranofin	Phase I trial: Phase I clinical trial results of auranofin, a novel antiparasitic agent	NCT02089048	Capparreli et al. (2016)
**Indirect-acting drugs**				
6	Doxycycline	Endosymbiotic bacteria in worms as targets for a novel chemotherapy in filariasis	None	Hoerauf et al. (2000)
		Depletion of *Wolbachia* endobacteria in *Onchocerca volvulus* by doxycycline and microfilaridermia after ivermectin treatment	None	Hoerauf et al. (2001)
		Doxycycline in the treatment of human onchocerciasis: kinetics of *Wolbachia* endobacteria reduction and of inhibition of embryogenesis in female *Onchocerca worms*	None	Hoerauf et al. (2003)
		*Wolbachia* endobacteria depletion by doxycycline as antifilarial therapy has macrofilaricidal activity in onchocerciasis: a randomized placebo-controlled study	ISRCTN 71,141,922	Hoerauf et al. (2008)
		Effects of ivermectin with and without doxycycline on clinical symptoms of onchocerciasis	None	Masud et al. (2009)
		Community-directed delivery of doxycycline for the treatment of onchocerciasis in areas of co-endemicity with loiasis in Cameroon	None	Wanji et al. (2009)
		Macrofilaricidal activity after doxycycline only treatment of *Onchocerca volvulus* in an area of *Loa loa* co-endemicity: a randomized controlled trial	ISRCTN48118452	Turner et al. (2010)
		Long term impact of large scale community-directed delivery of doxycycline for the treatment of onchocerciasis	None	Tamarozzi et al. (2012)
		Doxycycline leads to sterility and enhanced killing of female *Onchocerca volvulus* worms in an area with persistent microfilaridermia after repeated ivermectin treatment: a randomized, placebo-controlled, double-blind trial	ISRCTN 66,649,839	Debrah et al. (2015)
		Comparison of doxycycline, minocycline, doxycycline plus albendazole and albendazole alone in their efficacy against onchocerciasis in a randomized, open-label, pilot trial	ISRCTN 06,010,453	Klarmann-Schulz et al. (2017)
		Clinical trial of rifampin and azithromycin for the treatment of river blindness	NCT00127504	Not published
7	Rifampicin	No depletion of *Wolbachia* from *Onchocerca volvulus* after a short course of rifampin and/or azithromycin	None	Richards et al. (2007)
		Efficacy of 2- and 4-week rifampicin treatment on the *Wolbachia* of *Onchocerca volvulus*	None	Specht et al. (2008)
8	TylAMac (ABBV-4083)	Study to assess adverse events, change in disease activity and how oral ABBV-4083 capsules when given alone or in combination with albendazole capsules moves in the body of adult participants with *Onchocerca volvulus* infection	NCT04913610	Not yet started

*Used in the FDA registration package

**Table 2:** Major preclinical studies in onchocerciasis models

Drug name	Study title	Short description	Reference
Ivermectin	The microfilaricidal activity of ivermectin in vitro and in vivo	Ivermectin was tested against of *O. lienalis*, *B. pahangi* and *D. immitis* microfilariae in vitro and in vivo in the mouse	Devaney and Howels (1984)
	Evaluation of drugs against onchocerca microfilariae in an inbred mouse model	Ivermectin was tested against of *O. lienalis* microfilariae in vivo in the mouse	Bianco et al. (1986)
	Drug activity against *Onchocerca gutturosa* males in vitro: a model for chemotherapeutic research on onchocerciasis	Ivermectin was tested against *B. pahangi* and adult male *O. gutturosa.*The latter was considered as a suitable surrogate for *O. volvulus*	Townson et al. (1987)
	The development of a laboratory model for onchocerciasis using *Onchocerca gutturosa*: in vitro culture, collagenase effects, drug studies and cryopreservation	Ivermectin was tested against *O. gutturosa.* The latter was considered as a suitable surrogate for *O. volvulus*	Townson et al. (1987)
	Chemotherapy of *Onchocerca lienalis* microfilariae in mice: a model for the evaluation of novel compounds for the treatment of onchocerciasis	Ivermectin was tested against *O. lienalis* microfilariae injected into inbred CBA/Ca mice	Townson et al. (1988)
	In vitro effects of ivermectin on *Onchocerca volvulus* microfilariae assessed by observation and by inoculation into *Similium damnosum *sensu lato	Ivermectin was tested in vitro against *O. volvulus* microfilarae collected from the skin of a patients	Chavasse and Davies (1990)
	The effects of ivermectin used in combination with other known anti-parasitic drugs on adult *Onchocerca guuurosa* and *O. volvulus* in vitro	Ivermectin was tested in vitro, in combination with other antiparasitic drugs, against adult *O. gutturosa* and *O. volvulus* using MTT colorimetry and worm motility	Townson et al. (1990)
	The effects of ivermectin on the viability of *Onchocerca lienalis* microfilariae in vitro and on their subsequent development in the blackfly vector, *Simulium ornatum*	Ivermectin was tested in vitro against *O. lienalis microfilariae* using MTT colorimetry and motility	Townson and Tagboto (1991)
Ivermectin, moxidectin, oxfendazole and/or emodepside	*Onchocerca volvulus* and *O. lienalis:* the microfilaricidal activity of moxidectin compared with that of ivermectin in vitro and in vivo	Ivermectin and moxidectin were tested in vivo against *O. volvulus and O lienalis* microfilarae injected into inbred CBA/Ca mice	Tagboto and Townson (1996)
	Drugs that target early stages of *Onchocerca volvulus*: a revisited means to facilitate the elimination goals for onchocerciasis	Ivermectin, moxidectin, oxfendazole and emodepside and moxidectin were tested in vitro against *O. volvulus* L3 and L4 stages	Jawahar et al. (2021)
	Experimental chemotherapy of filariasis: comparative evaluation of the efficacy of filaricidal compounds in Mastomys coucha infected with *Litomosoides carinii*, *Acanthocheilonema viteae*, *Brugia malayi* and *B. pahangi*	Ivermectin and oxfendazole were tested in vivo in *Mastomys coucha* infected with adults and microfilariae *L. sigmodontis*, *A. viteae*, *B. malayi* or *B. pahangi*	Zahner and Schares (1993)
Oxfendazole	Oxfendazole mediates macrofilaricidal efficacy against the filarial nematode *Litomosoides sigmodontis* in vivo and inhibits *Onchocerca* spec. motility in vitro	Oxfendazole was tested in vitro against adult worms *O. gutturosa*, pre-adult *O. volvulus* and microfilariae *O. lienalis* In vivo activity was evaluated in gerbils infected with *L. sigmodontis*	Hübner et al. (2020)
	Development and validation of an *Onchocerca ochengi* adult male worm gerbil model for macrofilaricidal drug screening	Oxfendazole was tested in vivo against adult male worms *O. ochengi* transplanted in gerbils	Cho-Ngwa et al. (2019)
Emodepside	Emodepside targets SLO-1 channels of *Onchocerca ochengi* and induces broad anthelmintic effects in a bovine model of onchocerciasis	Emodepside was tested in vivo against adult worms *O. ochengi* in naturally infected cattels	Bah et al. (2021)
	Filaricidal efficacy of anthelmintically active cyclodepsipeptides	Emodepside was tested in vivo in *Mastomys coucha* infected with *L. sigmodontis*, *A. viteae* and *B. malayi*	Zahner et al. (2001)
	Effect of chemotherapeutic treatment on cytokine (IFN-gamma, IL-2, IL-4, IL-5, IL-10) gene transcription in response to specific antigens in *Brugia malayi*-infected *Mastomys coucha*	Emodepside was tested in vivo in *Mastomys coucha* infected with *B. malayi*	Saunders et al. (2008)
	Effects of Bay 44–4400, a new cyclodepsipeptide, on developing stages of filariae (*Acanthocheilonema viteae*, *Brugia malayi*, *Litomosoides sigmodontis*) in the rodent *Mastomys coucha*	Emodepside was tested in vivo in *Mastomys coucha* infected with *L. sigmodontis*, *A. viteae* and *B. malayi*	Zahner et al. (2001)
	Activity of the cyclooctadepsipeptide emodepside against *Onchocerca gutturosa*, *Onchocerca lienalis* and *Brugia pahangi*	Emodepside was tested in vitro against adult and worms *O. gutturosa*, *B. pahangi* and microfilariae *O. lienalis* and *B. pahangi* In vivo activity was evaluated in mice infected with *O. lienalis* microfilariae	Townson (2005)
Auranofin	Repurposing auranofin as a lead candidate for treatment of lymphatic filariasis and onchocerciasis	Auranofin was tested in vitro against adult and worms *O. ochengi*, *B. pahangi* and *B. malayi.* In vitro, auranofin was selective against microfilariae *O. ochengi* and *L. loa* In vivo auranofin was evaluated in gerbils infected with *B. pahangi*	Bulman et al. (2015)
Doxycycline and Rifampicine	Prophylactic activity of tetracycline against *Brugia pahangi* infection in jirds (*Meriones unguiculatus*)	Tetracycline inhibit the development of third-stage infective larvae (L3) of *B. pahangi* to adult worms in gerbils	Bosshardt et al. (1993)
	In vitro activity of antimicrobial agents against the endosymbiont *Wolbachia pipientis*	Doxycycline and rifampicin were tested against rickettsia-like bacteria of the genus *Wolbachia*	Hermans et al. (2001)
	Antibiotic therapy in murine filariasis (*Litomosoides sigmodontis*): comparative effects of doxycycline and rifampicin on *Wolbachia* and filarial viability	Doxycycline and rifampicin were tested in vivo in mice infected with *L. sigmodontis.* The redout of is depletion of Wolbachiality	Volkmann et al. (2003)
	Short-course, high-dose rifampicin achieves *Wolbachia* depletion predictive of curative outcomes in preclinical models of lymphatic filariasis and onchocerciasis	Rifampicin was tested in vivo in SCID mice infected with *B. malayi* or *O. ochengi.* The redout of is depletion of Wolbachiality	Aljayyoussi et al. (2017)
ABBV-4083	Discovery of ABBV-4083, a novel analog of Tylosin A that has potent anti-*Wolbachia* and anti-filarial activity	ABBV-4083 was tested in vitro against *Wolbachia*. Depletion of the endosymbiont *Wolbachia* was evaluated in vivo in mice infected with *L. sigmodontis*	Von-Geldern et al. (2019)
	In vivo kinetics of *Wolbachia* depletion by ABBV-4083 in *L*. *sigmodontis* adult worms and microfilariae	Depletion of the endosymbiont *Wolbachia* was evaluated in vivo in mice infected with *L. sigmodontis* Inhibition of embryogenesis was tested in gerbils infected with *L. sigmodontis*	Hübner et al. (2019a, b)
	Preclinical development of an oral anti-*Wolbachia* macrolide drug for the treatment of lymphatic filariasis and onchocerciasis	The efficacy of two tylosin A analogs (A-1535469 and A-1574083) against *Wolbachia* was tested in mice infected with *B. malayi*, *L. sigmodontis* or *O. ochengi*	Taylor et al. (2019)
AWZ-1066S	AWZ1066S, a highly specific anti-*Wolbachia* drug candidate for a short-course treatment of filariasis	AWZ1066S was tested in vitro against *Wolbachia* Depletion of the endosymbiont *Wolbachia* was evaluated in vivo in mice infected with *B. malayi* and gerbils infected *L. sigmodontis*. Inhibition of embryogenesis was tested in the latter	Hong et al. (2019)
	Development and validation of a high-throughput anti-*Wolbachia* whole-cell screen: a route to macrofilaricidal drugs against onchocerciasis and lymphatic filariasis	AWZ1066S was tested in vitro against *Wolbachia*	Clare et al. (2015)
Corallopyronin A (Cor A)	Corallopyronin A specifically targets and depletes essential obligate *Wolbachia* endobacteria from filarial nematodes in vivo	Cor A was tested in vitro against *Wolbachia* Depletion of the endosymbiont *Wolbachia* was evaluated in vivo in mice infected with *L. sigmodontis*	Schiefer et al. (2012)

### Direct-acting drugs

#### Ivermectin (IVM)

Historical chemotherapeutic treatments for onchocerciasis, such as suramin and DEC, were of limited efficacy and/or safety (Awadzi and Gilles 1992, Budden 1976; Duke 1968). Neither intervention was suitable for MDA, and both drugs have been removed from use for onchocerciasis. The situation changed with the introduction of IVM. *Streptomyces avermitilis*, a microbe that produces anthelmintic avermectins, was isolated from a soil sample obtained on a Japanese golf course in a project led by Satoshi Omura at the Kitasato Institute, and the commercial product IVM was then obtained and developed by a team led by William Campbell at Merck & Co. Both researchers were rightfully acknowledged with the Nobel Prize in Medicine in 2015 for the impact of their discovery on global health. IVM has been shown to be also active against human ectoparasites like lice and rabies (Youssef et al. 1995). Based on its activity against filarial parasites of veterinary significance, in particular *Dirofilaria immitis* (Campbell 1983), but also in other experimental animal models, IVM was tested in human patients with onchocerciasis and shown to be effective as a prolonged action microfilaricide after a single oral dose, with few side effects (see Table 1). Adult worms examined after nodulectomy were found to have prolonged suppression of microfilarial production. In the mid-1980s, IVM became the first-choice drug for onchocerciasis, due to its safety profile in microfilaremic patients. Several studies led to its registration, comparing its efficacy against DEC and identifying the reasonable dose to be used in MDA (Greene et al. 1985; Lariviere et al. 1985; Diallo et al. 1986; Awadzi et al. 1986; White et al. 1987). The results suggested that there is no advantage in administering doses > 150 μg/kg and that the higher dose of 200 μg/kg may be associated with an increased incidence of adverse effects. Additional data and a longer follow-up period would be needed to clearly assess the performance of this dose compared with 100 μg/kg. Thus, 150 μg/kg was chosen as the dose of IVM for use in onchocerciasis control programmes. A higher dose (800 μg/kg) was tested for macrofilaricidal efficacy; IVM at 150 μg/kg or 800 μg/kg given every 3 months had some macrofilaricidal effects, but of insufficient magnitude to warrant incorporation into MDA programs (Gardon et al. 2002).

The realization that a single yearly treatment with IVM could markedly limit transmission and pathology led to the decision by Merck & Co. to donate the drug through the Mectizan Donation Program for MDA to control onchocerciasis. Current guidelines recommend annual (or biannual) treatment of every individual in targeted communities with an oral tablet of IVM (Mectizan), based on height. The current height-based IVM dosing has a range of 3–12 mg for four different height groups (90–119 cm, 120–140 cm, 141–158 cm and > 158 cm). Children below 15 kg and visibly pregnant women are excluded from MDA (Crompton 2006). Recent meta-analysis further suggests that IVM is safe in children weighing less than 15 kg (Jittamala et al. 2021).

The recognition that SAEs, including coma and death, can occur in individuals harbouring high levels of *L. loa* microfilaremia who are treated with IVM (Boussinesq et al. 2003; Chesnais et al. 2020) has hindered the extension of MDA programs to loiasis endemic regions since individual diagnosis of *L. loa* microfilaremia using microscopic assays would have been required. A new strategy termed “Test and Not Treat” (TaNT), based on the use of the LoaScope to estimate *L. loa* microfilaraemia before treatment to exclude heavily infected patients, could allow safe implementation of MDA in loiasis endemic areas (Kamgno et al. 2017).

#### Moxidectin

Moxidectin belongs to the milbemycin family of antiparasitic endectocides, which were isolated in 1967 following fermentation of *Streptomyces hygroscopes* (a soil bacterium). In 1972, the 16-membered macrocyclic lactone structure of milbemycin was elucidated. In 1983, nemadectin (F-29249α), an active milbemycin product, was isolated from the fermentation of *Streptomyces cyaneogriseus*. Moxidectin was later chemically derived from nemadectin by the addition of a methoxime moiety at carbon 23 (Prichard and Geary 2019). The mechanism of action of moxidectin is thought to be similar to that of IVM, mediated through increased permeability of glutamate-gated chloride channels (GluCls) resulting in an influx of chloride ions and subsequent paralysis/death of the parasite (Wolstenholme et al. 2016). Treatment of mice with a single dose of moxidectin at 15 or 1.5 μg/kg resulted in mf reductions of 96% and 23%, respectively. In contrast, a 48% reduction was observed with a single dose of 15 μg/kg IVM and a 2% increase with 1.5 μg/kg IVM/kg (Tagboto and Townson 1996). Moxidectin is minimally metabolized, has low affinity to p-glycoprotein transporters and shows an extended plasma half-life of 20–43 days in humans compared to IVM (Korth-Bradley et al. 2012; Prichard et al. 2012). This is because moxidectin is more lipophilic than IVM, which may result in greater retention in adipose tissue (Prichard et al. 2012). Moxidectin is effluxed by P-gp transporters at the blood–brain barrier (Kiki-Mvouaka et al. 2010), suggesting lower neurotoxicity for moxidectin compared to IVM, as recently observed in experimental animals (Janko and Geyer 2013). In people infected with *O. volvulus*, moxidectin was able to reduce and maintain low skin microfilarial density for longer than IVM (Awadzi et al. 2014). Also, moxidectin has been shown to have activity against human scabies (Mounsey et al. 2016).

Following a controlled, double-blind phase 3 trial conducted in Ghana, Liberia and the Democratic Republic of the Congo, moxidectin was registered for the treatment of onchocerciasis and is recognized as a highly efficacious microfilaricide (Opoku et al. 2018). If donated, this molecule could be an alternative to IVM in MDA programs (Milton et al. 2020). The Medicines Development for Global Health (MDGH) is currently starting recruitment for three clinical trials evaluating safety in a large patient cohort, paediatric dose-finding and comparison of moxidectin and IVM in annual and biannual dosing (see Table 1).

#### Emodepside

The semi-synthetic anthelminthic emodepside (synonyms: PF1022-221, BAY 44–4400 or bismorpholino-cyclooctadepsipeptide) and its parent fermentation product PF1022A are members of the N-methylated cyclooctadepsipeptides, derived from a fungus (*Rosellinia* sp. PF1022). Emodepside is characterized by two morpholine rings in the para-position of each of the two (R)-phenyllactic acids, which increase solubility and improve the bioavailability of the compound compared to its natural precursor PF1022A (Harder et al. 2005). Emodepside is registered as a combination product with praziquantel (Profender®) for the treatment of cats and dogs infected with, or at risk of, infection with nematodes and cestodes, and as a combination product with toltrazuril (Procox®) for the treatment of puppies with demonstrated or suspected infection with nematodes and coccidia.

Several targets have been suggested for the cyclooctadepsipeptides, with the voltage-gated, calcium-activated potassium channel SLO-1 as most likely candidate (Kulke et al. 2014). Additionally, the G-protein coupled receptor LAT-1 (Saeger et al. 2001) and ionotropic GABAA receptors (Chen et al. 1996; Miltsch et al. 2012) might contribute to susceptibility to cyclooctadepsipeptides.

Action on the SLO-1 K channel results in an inhibitory effect on motility, pharyngeal pumping and egg laying of nematodes. In addition to emodepside’s success as a veterinary anthelminthic, several experiments showed its effect on filarial nematodes in various models, including initial studies on *L. sigmodontis* in *Mastomys* (Zahner et al. 2001) and recently *O. ochengi* in cattle (Bah et al. 2021). These experiments showed macrofilaricidal efficacy after 5 consecutive doses of 5–100 mg/kg in rodents and 0.75 mg/kg in cattle. Emodepside has a broad activity against different filarial species and stages including L3, L4, adult and mf of filarial parasites (reviewed in Krücken et al. 2021).

Emodepside is currently being evaluated for the treatment of human onchocerciasis within the scope of a drug development partnership between the Drugs for Neglected Diseases *initiative* (DND*i*) and Bayer AG (Krücken et al. 2021). Based on such promising results from preclinical studies, a phase I package (single and multiple ascending dose studies, food effect and relative bioavailability studies) was conducted in healthy volunteers (Gillon et al. 2021, Table 1). These studies showed that effective doses based on experiments in rodents and cattle can be reached in humans. Bayer AG has further developed a formulation with significantly improved biopharmaceutical properties and higher bioavailability compared to the conventional standard immediate release crystalline tablet (see Table 1). DND*i* plans to start a proof-of-concept trial later in 2021 to determine the efficacy of emodepside in patients with onchocerciasis (see Table 1).

#### Oxfendazole

Oxfendadazole, [5-(phenylsulphinyl)-1H-benzimidazole-2-yl] carbamic acid methyl ester, is a broad-spectrum benzimidazole anthelminthic used for veterinary practice against lungworms and enteric helminths in beef livestock. It has been established that benzimidazoles selectively bind with high affinity to parasite β-tubulin and inhibit microtubule polymerization, resulting in the destruction of cell structure and subsequent death of the parasite (Lacey 1990). Oxfendazole has gained much interest recently and is being repurposed for human use against tissue-dwelling larval helminths after demonstrating moderate macrofilaricidal activity in vitro against the adult stages of *O. volvulus* and *Onchocerca gutturosa*. In vivo oral treatment with oxfendazole for 5 days and 10 days in the murine *Litomosoides sigmodontis* model provided 100% macrofilaricidal clearance of the parasite and inhibited filarial embryogenesis in patent *L*. *sigmodontis*-infected jirds, respectively but had no direct effect on the mf (Hübner et al. 2020). Such an efficacy profile is desirable for MDA, because severe pathologies associated with dead microfilariae would be avoided.

As oxfendazole has broad antihelmintic activity through inhibition of tubulin polymerization, it is open for debate whether it should be evaluated as a potential drug against loiasis, a highly neglected NTD, targeting the adults but not the mf.

The human efficacious dose predicted by allometric scaling ranges from 1.5 to 4.1 mg/kg (Hübner et al. 2020). This dose has been proven to be safe in phase 1 clinical trials, including a multiple ascending dose study using five daily doses of 3, 7.5 and 15 mg/kg (Table 1) (Bach et al. 2020) and a single ascending dose phase 1 study in humans (An et al. 2019). DND*i* has taken oxfendazole into their portfolio as a candidate for development for onchocerciasis (https://www.dndi.org/diseases-projects/portfolio/oxfendazole/). A bioavailability study to test the exposure of a tablet for field use is currently planned within the HELP consortium activities of the Horizon 2020/EU project (www.eliminateworms.org). The aim of the HELP consotrtium is to advance existing drug candidates along the development pipeline to fuel the empty anthelminthic drug pipeline for soil-transmitted helminths and onchocerciasis, joining expertise from both areas.

#### Auranofin

Auranofin [2,3,4,6-tetra-*o*-acetyl-l-thio-β-d-glycopyranp-sato-*S*-(triethyl-phosphine)-gold] is an FDA-approved drug that was originally developed to treat rheumatoid arthritis in humans in 1985. Previous studies have shown that this drug is effective against several parasites; recently, Bulman et al. (2015) found that auranofin was effective in vitro in killing *O. ochengi* adult worms and in inhibiting the moulting of *O. volvulus* L3s with IC_50_ of 3 and 0.3 μM, respectively. The in vivo efficacy of auranofin was tested in gerbils infected with *Brugia pahangi*; a dosing regimen of 5 mg/kg *bis in die* (BID) on weekdays and *semil in die* (SID) on weekends was applied for 28 days (for a total of 48 doses). A 91% reduction of adult worm burden was observed in the auranofin-treated group compared to the control group. Evidence in several species of parasites suggests that thioredoxin reductase and a similar enzyme, thioredoxin glutathione reductase, are targeted by auranofin (Tejman-Yarden et al. 2013). Phase I first in human studies have been carried out and it has been shown to have a half-life (*t*_1/2_) of 35 days (Table 1), meaning that steady-state blood levels could be reached in long-term therapy for onchocerciasis, a chronic disease (Capparelli et al; 2016).

#### Flubendazole (FBZ)

As a member of the benzimidazole anthelmintic class, FBZ is a tubulin inhibitor with aneugenic and embryotoxic effects like those observed with albendazole and other benzimidazole anthelmintics have been reported (Tweats et al. 2016; Longo et al. 2014). It has been shown in animal models and in a human trial (Dominguez-Vazquez et al., 1983) that parenterally administrated FBZ can attain 100% macrofilaricidal efficacy (Mackenzie and Geary, 2011). For decades, WHO and TDR have been advocating for the development of macrofilaricidal treatments for the elimination of onchocerciasis. This prompted the re-evaluation of FBZ as a potential macrofilaricide. However, because of its poor solubility and bioavailability, administration of FBZ via the oral route provides only marginal systemic exposure (Michiels et al. 1982). As parenteral administration of FBZ is not compatible with MDA programs, the initial goal of the project was to develop a new orally available formulation providing sufficient systemic exposure and high macrofilaricidal efficacy with short dosing regimens (Mackenzie and Geary 2011).

This aim was the driving force behind the decision by the Bill and Melinda Gates Foundation (BMGF) to fund preclinical development work with FBZ under the auspices of DND*i*, which led to the development of an amorphous solid dispersion (ASD) formulation first by AbbVie and then by Janssen Pharmaceutica. FBZ formulated as an ASD is orally bioavailable and was used for initial toxicological investigations (Longo et al. 2014; Tweats et al. 2016). Following encouraging in vivo efficacy results, Janssen Pharmaceutica further evaluated the orally available ASD formulation of FBZ and demonstrated macrofilaricidal in vivo efficacy (Fischer et al. 2019; Sjoberg et al. 2019; Hübner et al. 2019a, b)*.* Despite these encouraging results, efficacious concentrations were associated with toxicity. Following consultation with FDA, Janssen Pharmaceutica decided to discontinue the development of FBZ because the risk–benefit ratio for patients was not considered to be favourable (Lachau-Durand et al. 2019).

### Indirect-acting drugs

#### Doxycycline

Doxycycline is a broad-spectrum antibiotic belonging to the tetracycline family and is included in the World Health Organization’s list of essential medicines (WHO 2019). It inhibits the production of bacterial proteins by binding to the 30S ribosomal subunit, thus preventing the binding of transfer RNA to messenger RNA and further replication of the bacteria (Chopra and Roberts 2001). Bosshardt and colleagues were the first to show that tetracycline treatment of jirds infected with *Brugia pahangi* was able to inhibit the development of third-stage larvae into adult worms and the development of microfilaremia (Bosshardt et al. 1993). Many experiments followed, exploring the effect of *Wolbachia* depletion in various parasite/host combinations (Wan et al. 2019). Since doxycycline was already a registered tetracycline, it did not take long to conduct studies in humans infected with onchocerciasis, providing proof-of-concept for the therapeutic effect of *Wolbachia* depletion. Initially, a treatment of 100 mg/day for 6 weeks was chosen, which resulted in the inhibition of microfilaridermia over the observation period of 18 months (Hoerauf et al. 2001). A number of studies followed, investigating other dose regimens (see Table 1). It is important to note that this indirect mode of action, via the inhibition of embryogenesis, results in a slow decline of mf in the infected human and prevents the adverse reactions (Mazzotti reaction) seen when there is a rapid reduction of mf, for example after DEC treatment. It has been further argued that the absence of bacterial antigens per se avoids inflammatory reactions associated with the killing of macro- or microfilariae (Taylor et al. 2010).

There was clear evidence for the macrofilaricidal activity of doxycycline when longer follow-up time points (20–24 months) were chosen (Hoerauf et al. 2008). Doxycycline’s mode of action allows its use in areas coendemic with Loiasis since *Loa loa* does not harbour *Wolbachia* (Grobusch et al, 2003). The use of doxycycline is, however, clearly contraindicated for large-scale treatment programs because of logistical challenges, the lengthy course of treatment (4–6 weeks), contraindications in children under 8 years and in pregnancy (Hoerauf et al. 2001). This gave the starting signal to setup the AWOL consortium (Taylor et al. 2014) to identify drugs and regimens that reduce the treatment period and which would be safe in currently excluded target populations (children and pregnant women) using a preclinical screening cascade developed by the consortium partners (Johnston et al. 2014; Specht et al. 2018) (see Table 1). Several promising results from either single treatments or combinations have been or are currently being, further explored, such as the new chemical entity TylAMac as well as registered drugs, such as minocycline and a rifampicin and moxifloxacin combination.

#### Rifampicin

Rifampicin is a semi-synthetic antibiotic produced from *Streptomyces mediterranei.* Its mechanism of action is through inhibition of DNA-dependent RNA polymerase activity by forming a stable complex with the enzyme, thus suppressing the initiation of RNA synthesis in *Wolbachia* (Campbell et al. 2001). The in vitro activity obtained in the *Wolbachia*-infected C6/36 cell (C6/36 wAlbB) assay for rifampicin exhibited an EC_50_ of 1.3 nM (approximately 16.2-fold more potent than doxycycline). The effect of rifampicin evaluated in SCID mice infected with *Onchocerca ochengi* and *Brugia malayi* showed a dose-dependent activity against *Wolbachia*. When administrated at 25 mg/kg bid for 7 days, rifampicin displayed significantly superior anti-*Wolbachia* activity against *Brugia malayi* compared to a 4-week doxycycline treatment at the same dose (Aljayyoussi et al. 2017).

Rifampicin was administrated for 14 days daily at 50 mg/kg orally to BALB/c mice infected with the rodent filaria, *Litomosoides sigmodontis*, and compared to doxycycline administrated orally at 25 mg/kg for 21 days. Depletion of endobacteria was observed following treatment with rifampicin but not following treatment with doxycycline. Furthermore, treatment with rifampicin resulted in a significant reduction in filarial survival, size and fertility (Volkmann et al. 2003). However, clinical studies in humans have shown that rifampicin 10 mg/kg/day administered for 2 or 4 weeks has an inferior activity to 6-week doxycycline in onchocerciasis (Specht et al. 2008; Richards et al. 2007).

In SCID mice, a dose of 35 mg/kg administrated for 14-days elicits a reduction in *Wolbachia* above 90%. Using pharmacokinetic/pharmacodynamic modelling, this dosing regimen corresponds to a human equivalent dose of 30–40 mg/kg. It has been suggested that this clinical dosing regimen could be effective for the treatment of onchocerciasis (Aljayyoussi et al. 2017).

#### TylAMac (ABBV-4083)

The discovery of ABBV-4083 began with the screening of a representative set of the AbbVie antibiotics collection (129 compounds). This focused library was tested against *Wolbachia pipientis* in an insect cell line within the AWOL consortium. From this screen, Tylosin A (TylA), a commercial veterinary macrolide antibiotic, was identified as novel lead. TylA is a macrolide antibiotic that is active mostly against Gram-positive bacteria and mycoplasmas by binding to the 50S ribosomal subunit, inhibiting protein synthesis (Risch et al. 2021). TylA has displayed potent anti-*Wolbachia* activity with an in vitro EC_50_ of 28 nM, but TylA is characterized by poor oral bioavailability resulting from low permeability (< 0.1·10^−6^ cm/s) in a canine kidney cell monolayer system (MDR-MDCK). Therefore, improving drug absorption by increasing permeability became a primary goal for lead optimization. Derivatization of the 4″-OH group of TylA (on the mycaminose sugar) was carried out by AbbVie to improve oral absorption while simultaneously increasing anti-*Wolbachia* potency. Further optimization of this substituent led to the development of ABBV-4083, the 4″-(4F-benzyl) stable analogue, which is very active against *Wolbachia*, with an improved pharmacokinetic profile. ABBV-4083 was evaluated in gerbils orally at 150 mg/kg, once daily for 14 days. Even 16 weeks post-treatment initiation (PTI), *Wolbachia* levels remained reduced by > 99.9% in the female adult worms recovered from the host animals. Furthermore, 7 weeks PTI, levels of circulating microfilariae declined and were completely cleared from 12 weeks PTI (von Geldern et al. 2019). The efficacy of A-1574083 (now called ABBV4083) was tested in mouse and gerbil infection models of lymphatic filariasis (*Brugia malayi* and *Litomosoides sigmodontis*) and onchocerciasis (*Onchocerca ochengi*). A 1- or 2-week course of oral A-1574083 provided > 90% *Wolbachia* depletion from nematodes in infected animals, resulting in a block in embryogenesis and depletion of microfilarial worm loads with superior efficacy compared to a 3- to 4-week course of doxycycline or minocycline (Taylor et al. 2019) (Table 2). Following completion of regulatory enabling studies and first in human clinical trials, DND*i* is currently preparing a proof-of-concept trial later in 2021 to determine the efficacy of ABBV-4083 in onchocerciasis patients.

#### AWZ-1066S

To identify novel anti-*Wolbachia* chemotypes, 10,000 compounds selected from a commercial library were screened using a phenotypic cell-based screen incorporating a *Wolbachia*-infected cell line. Identified hits were optimized for both anti-*Wolbachia* activity and drug metabolism/pharmacokinetic (DMPK) properties. The core of one of the identified hits was thienopyrimidine. Optimization of this starting hit molecule led to AWB158 with a quinazoline core, which was further optimized into AWZ1066 with an azaquinazoline core. These modifications have resolved the metabolic stability associated with the original core while improving potency against *Wolbachia*. AWZ1066 has two enantiomers, namely AWZ1066S and AWZ1066R. The (S)-isomer is more potent compared to the (R)-isomer both in in vitro anti-*Wolbachia* assays (EC_50_ = 2.5 ± 0.4 vs. 14.4 ± 3.7 nM) and in a microfilaria assay (121 vs. 408 nM). AWZ1066 (racemic mixture) was active against *Wolbachia* (Clare et al. 2015) with an EC_50_ of 2.6 ± 0.5 nM. Despite being a mammalian P-gp transporter substrate (i.e. it is pumped out of the cell to be eliminated), AWZ1066S showed good oral bioavailability (range 54 to 91%) across a range of dosages up to 250 mg/kg in *Brugia malayi* SCID CB.17 mice and gebril model. Treatment with AWZ1066S administrated twice-daily at 100 and 50 mg/kg orally for 7 days in a *Litomosoides sigmodontis* gerbil model was investigated. The *Wolbachia* load was reduced by > 99% compared to the untreated group. Fourteen weeks post-treatment, microfilaremia was inhibited in treated animals. AWZ1066S also has a faster kill rate compared with other known antibiotics tested against *Wolbachia* in vitro and its mode of action is by inhibition of protein synthesis. AWZ1066S can achieve maximum reduction of *Wolbachia* just after 1 day of drug exposure compared with the other antibiotics tested (1 to 6 days of exposure) (Hong et al. 2019). A global health innovative fund has been awarded for the development of AWZ1066S, a small molecule anti-*Wolbachia* candidate macrofilaricide drug (https://www.prnewswire.com/news/global-health-innovative-technology-%28ghit%29-fund/).

#### Corallopyronin A

Corallopyronin A (CorA) is a natural compound originally isolated from the myxobacterial strain *Corallococcus coralloides* c127, representing a new structural type of antibiotic having a pyrone ring conferring rigidity to the central part of the molecule with two chains attached to the ring. One side of the chain carries a terminal vinyl carbamate functionality, while the other is characterized by several double bonds and a hydroxyl group (Schiefer et al. 2012). Both groups interact with the bacterial enzyme RNA polymerase (RNAP), preventing the correct interaction with the DNA template (Belogurov et al. 2009). Because the site and mode of action of CorA are different from that of rifampicin, CorA has activity against rifampicin-resistant *S. aureus* (O’Neill et al. 2000). Eukaryotic RNAP is resistant to CorA (Irschik et al. 1985), making this antibiotic attractive for further studies.

Using the C6/36 cell line from *Aedes albopictus* infected with *Wolbachia pipientis*, CorA depleted *Wolbachia* in a dose-dependent manner, and 1 μg/mL (1.895 μM) of Cor depleted the endobacteria from the cells to levels equivalent to those of 4 μg/mL (7.8 μM) doxycycline and 0.1 μg/mL (121.5 nM) rifampicin. Assessment of 4-week treatment of 35 mg/kg/day CorA in mice infected with *L. sigmodontis* showed a blockage of worm development at the L4 stage and thus reduced worm length, sustained microfilaria clearance and inhibited embryogenesis; CorA showed macrofilaricidal efficacy during chronic filarial infection (Schiefer et al. 2012). A combination therapy of CorA and albendazole further enhanced the macrofilaricidal efficacy, with a shortened treatment regimen of 7, 10 and 14 days at a CorA dose as low as 10 mg/kg. This is the first anti-wolbachial treatment that has been able to achieve robust macrofilaricidal efficacy in the *L*. *sigmodontis* model (Schiefer et al. 2020). CorA is being developed for clinical phase 1 trials within the German Center for Infection Research (https://www.dzif.de/en/corallopyronin-new-antibiotic-against-worm-infections) and the HELP consortium within Horizon 2020/EU (www.eliminateworms.org).

## Conclusion

It is worth noting that programs have made tremendous efforts towards the control of onchocerciasis using IVM as the sole prophylactic microfilaricidal therapy. For elimination to be achieved at a faster rate, suitable, safe and cost-effective macrofilaricides must be developed, and this is one of the top priority goals of the Drugs for Neglected Diseases *initiative*, which is developing emodepside, tylamac and oxfendazole. These potential macrofilaricides will help to interrupt the parasite life cycle, thus preventing the adult females from producing young larvae. However, drug development is handicapped by high attrition rates, and many promising molecules fail during preclinical development or in subsequent toxicological, safety and efficacy testing; thus, R&D costs in the aggregate are very high. The level of investment into R&D for new products for NTDs, as reported in the annual Global Funding of Innovation for Neglected Diseases (G-FINDER) surveys, shows that few NTD areas receive anywhere near the level of funding required and that funding, when it is available, is rarely allocated in a manner likely to move products through the pipeline to patients. Therefore, no dedicated drug development pipeline for human filariasis is in place and it is essential that stakeholders, funders, industry, academics and NGOs adopt a cooperative approach and share the responsibility to reduce risks and overcome these obstacles. Joint efforts are being made to cut the cost of R&D for new drugs for NTDs and increase the attractiveness of this sector to funders and investors. Supportive programs by the FDA (priority voucher program: https://www.fda.gov/about-fda/center-drug-evaluation-and-research-cder/tropical-disease-priority-review-voucher-program) and EMA (article 58: https://www.ema.europa.eu/en/human-regulatory/marketing-authorisation/medicines-use-outside-european-union) aim to increase incentives for companies to engage in drug development for NTDs. Furthermore, The 2012 London declaration (https://unitingtocombatntds.org/london-declaration-neglected-tropical-diseases/) united pharmaceutical companies, donors, endemic countries and non-government organizations in the recognition that new drugs need to be developed to achieve the elimination goals. The signatories of the London Declaration made a clear statement that it is important to take the initiative now for the development of new drugs to avoid a scenario in 2030, when elimination targets may not be achieved and valuable time wasted. Joint efforts such as in product development partnerships are necessary to achieve this goal. The result of such collaboration with pharmaceutical companies has led to the development of emodepside (Bayer AG, Bayer Animal Health; Krücken et al. 2021) and ABBV-4083 (AbbVie; Kempf and Marsh, 2020).

## Data Availability

Not applicable.
